# Who participates? Who frequents? Exploring the correlates of sports participation among Chinese adults: evidence from national survey

**DOI:** 10.3389/fpsyg.2025.1621125

**Published:** 2026-01-09

**Authors:** Yizhen Chao, Yaqing Wang, Zhenzhan Chang

**Affiliations:** 1 The Wushu Association of Dengzhou City, Dengzhou, Henan, China; 2 Department of Biochemistry and Biophysics, School of Basic Medical Sciences, Peking University, Beijing, China

**Keywords:** China, adults, correlates, health behavior, non-communicable diseases, physical activity, public health promotion, sports participation

## Abstract

**Background:**

Although the benefits of sports participation (SP)—such as reduced anxiety and depression and improved physical health—are well-established, physical inactivity continues to rise globally. In China, the proportion of adults regularly participating in sports remains low, underscoring the need for targeted interventions. However, research on the correlates of SP among Chinese adults is scarce. Given that correlates may differ across countries and between specific participation behaviors, it is essential to differentiate between types of participation to conduct a comprehensive analysis of the correlates of SP among Chinese adults. The objective of this study is to incorporate a broad range of independent variables validated in prior literature to conduct a comprehensive analysis of sports participation among Chinese adults, thereby providing empirical evidence for precise intervention strategies and future research directions.

**Methods:**

Using data from the 2021 China General Social Survey (CGSS), this study included 22 independent variables previously validated as significantly associated with SP. Three outcome variables of SP among Chinese adults were analyzed in this study: (1) whether participation, (2) participation frequency, and (3) frequent participation. The full dataset (*n* = 5,581) was used to examine the correlates of whether to participate, while a subsample of sports participants (*n* = 3,491) was used for the other two outcomes. Analyses involved descriptive statistics, univariate analysis, and backward stepwise regression.

**Results:**

Twelve variables—including provincial economies, settlement type, education, BMI, health issues influence, depression, internet access, watch competition, social class, economic status, car ownership, and age—were significantly associated with whether participation (all *p* < 0.05 across all regression models). Seven variables, including provincial economies, settlement type, health issues influence, depression, migration, children, and age, were significantly associated with both participation frequency and frequent participation (all *p* < 0.05 across all regression models).

**Conclusion:**

This study provides innovative insights into the correlates of SP among Chinese adults, with age, gender, depression, and migration showing patterns that differ from existing literature. While participation frequency and frequent participation show similar correlates, they differ significantly from the correlates of whether participation, highlighting the need for differentiated intervention strategies.

## 1 Introduction

Sports participation (SP) refers to an individual's engagement in any form of bodily movement performed by skeletal muscles that results in an increase in energy expenditure (World Health Organization, 2019). Common types of activities include running, dancing, swimming, and yoga (World Health Organization, 2019). The benefits of SP are well-documented. SP—across all intensities—has been shown to positively influence health-related quality of life, and both moderate-to-vigorous exercise and low-intensity activities such as walking are associated with reduced all-cause mortality risk (Barakou et al., 2025; Ekelund et al., 2019; Jakicic et al., 2019; Saint-Maurice et al., 2018). Meanwhile, substantial evidence indicates that moderate-to-vigorous SP contributes to reduced symptoms of anxiety and depression, while also improving cardiovascular and muscular health (Piercy et al., 2018). Notably, health benefits can be derived regardless of how SP is accumulated—even brief bouts as short as 5 min can be beneficial (Ekelund et al., 2019; Jakicic et al., 2019; Saint-Maurice et al., 2018). Encouragingly, recent studies suggest that even occasional low-frequency participation in sports may mitigate the cardiovascular risks associated with prolonged sedentary behavior (Koemel et al., 2025). Beyond physiological advantages, participation in sports fosters psychological resilience, which in turn enhances individuals' capacity to overcome life challenges and pursue long-term goals (Nothnagle and Knoester, 2025).

Despite the well-established benefits of SP, global inactivity rates are rising, exacerbating the burden of preventable diseases and premature mortality (Rouyard et al., 2025). Authoritative literature indicates that in 2022, 31.3% of adults worldwide were insufficiently physically active, compared to 23.4% in 2000 and 26.4% in 2010 (Rouyard et al., 2025). Meanwhile, physical inactivity and the prevalence of non-communicable diseases are particularly prominent in low- and middle-income countries (Rouyard et al., 2025). In response, the World Health Organization launched the Global Action Plan on Physical Activity 2018–2030, aiming to reduce global levels of physical inactivity by 15% by 2030 (World Health Organization, 2019).

As one of the most populous country in the world, China plays a pivotal role in the realization of global physical activity goals (United Nations Population Division, 2024). In alignment with the WHO initiative, the Chinese government introduced the “Healthy China 2030” blueprint (State Council of the People's Republic of China, 2016) and the “Healthy China Action Plan (2019–2030) (Healthy China Action Promotion Committee, 2019),” which set a clear target for SP: by 2030, at least 40% of the population should engage in regular sports (Healthy China Action Promotion Committee, 2019). Yet, recent surveys indicate that only 30.3% of Chinese adults meet this standard (National Physical Fitness Monitoring Center of the People's Republic of China, 2021). To bridge this gap, it is imperative to develop comprehensive and evidence-based interventions aimed specifically at promoting SP among Chinese adults. This requires precise analysis of the correlates of SP within this population.

Currently, research examining the determinants of SP among Chinese adults remains limited. Furthermore, existing literature demonstrates that the correlates of SP may vary across countries (e.g., Spain vs. the United Kingdom) (Kokolakakis et al., 2012) and also differ depending on how SP is measured (e.g., whether participation vs. participation frequency) (Oliveira-Brochado et al., 2017). Therefore, this study proposes two scientific hypotheses: (1) variables previously identified as significant predictors of SP in international literature will also be significantly associated with SP among Chinese adults, but the effect values may differ; and (2) the correlates of different measurement standards in SP are different. To comprehensively and accurately examine the correlates of SP among Chinese adults, this study is grounded in clear scientific hypotheses and follows a twofold analytical strategy. First, a wide range of independent variables previously validated in the literature as significantly associated with SP were included. Second, a detailed distinction is made between the SP of Chinese adults.

In conclusion, the purpose of this study is to incorporate a wide range of independent variables that have been validated in prior literature to be significantly associated with SP to conduct a comprehensive analysis of Chinese adults' SP. It aims to provide an objective basis and direction for developing precise interventions for Chinese adults' SP and to guide future research.

## 2 Methods

### 2.1 Data source

This study is a cross-sectional secondary data analysis based on national survey data. This study uses data from the 2021 Chinese General Social Survey (CGSS), a nationally representative, large-scale social survey led by Renmin University of China (Renmin University of China, 2023). The CGSS questionnaire comprises multiple thematic modules covering demographic characteristics, socioeconomic status, health conditions, behavioral patterns, and social attitudes. Numerous authoritative research publications have utilized CGSS data for professional academic studies (Zhong et al., 2022; Li et al., 2023; Chao et al., 2025), and the validity and reliability of this data are widely recognized. The 2021 CGSS is a nationally representative, large-scale continuous cross-sectional survey using a multistage stratified sampling design to collect data on Chinese residents aged 18 and above. The 2021 dataset comprises 8,148 valid samples and 700 variables. It provides extensive information suitable for analyzing SP. Its broad scope and data quality make it a reliable source for examining correlates of SP among Chinese adults. Detailed questionnaire items and response categories are available in the official 2021 CGSS Survey Manual. For more information and details about CGSS, please visit its official website: http://cgss.ruc.edu.cn/English/Home.htm.

This study utilizes anonymized and publicly available data. According to the provisions of Articles 1 and 2 under Clause 9 of the interpretation of the “Measures for Ethical Review of Life Sciences and Medical Research Involving Human Subjects” issued by the Department of Science, Technology, and Education of the National Health Commission of the People's Republic of China on February 27, 2023, this study is exempt from ethical review (Department of Science, 2023).

### 2.2 Variables measurement

#### 2.2.1 Outcome variables measurement

Previous research on SP has commonly included both whether participation and participation frequency as outcome variables (Borgers et al., 2016; Kellstedt et al., 2021; Eime et al., 2015). Following this practice, the present study also adopts these two variables. Evidence suggests that frequent participation in physical activities leads to significantly greater health improvements compared to low-frequency participation (Bailey and Brooke-Wavell, 2010; Kell and Rula, 2019; Kemmler and von Stengel, 2013). Additionally, official Chinese publications such as the General Administration of Sport of the People's Republic of China (2017) and the Healthy China Action Promotion Committee (2019) explicitly stipulate that Chinese adults who engage in at least three sessions of moderate-intensity physical activity lasting 30 min or more per week can be classified as regular exercisers (Healthy China Action Promotion Committee, 2019; General Administration of Sport of the People's Republic of China, 2017). To comprehensively investigate the correlates of SP among Chinese adults, this study additionally includes “frequent participation” as a third outcome variable. Participants who may meet the national recommendations are classified as high-frequency participants, while those who definitely do not meet these criteria are classified as low-frequency participants. Therefore, the three outcome variables in this study are: (1) whether participation, (2) participation frequency, and (3) frequent participation. The outcome variable “whether participation” refers to whether the research subjects engage in physical exercise. The outcome variable “participation frequency” refers to the frequency with which the research subjects engage in physical exercise. The outcome variable “frequent participation” refers to whether the research subjects are likely to achieve high-frequency physical exercise participation.

When analyzing factors associated with whether participation, the entire sample was utilized. However, analyses of participation frequency and frequent participation were only conducted in the subsample that reported participating in SP. For the analyses of participation frequency and frequent participation, a purposive subsampling approach was applied by restricting the analytic sample to sport participants. This approach is based on prior literature suggesting a fundamental distinction between individuals who never participate in sports and those who do, implying that the correlates differ substantially (Oliveira-Brochado et al., 2017; Eime et al., 2015). Accordingly, participants who reported no SP were excluded from analyses of participation frequency and frequent participation, and only the SP subsample was used for these analyses. The outcome variables, including whether participation, participation frequency, and frequent participation, were derived from the CGSS 2021 item A30.9. This question has been widely adopted in previous CGSS-based studies to assess sports participation (Zhong et al., 2022; Chao et al., 2025). Detailed information on the original question wording, response options and coding procedures is provided in Table 1.

**Table 1:** Measurement and assignment of all variables.

**Variable names**	**Questions in the original questionnaire**	**Original options**	**Measurement methods**	**Options after measurement**
Whether participation	A30.9 In the past year, did you regularly participate in physical activity during your free time?	1 =daily, 2 =several times a week, 3 =several times a month, 4=several times a year or less, 5= never	1, 2, 3 and 4 = 1 participate, 5 = 0 does not.	1 = yes, 0 = no
Frequent participation	A30.9 In the past year, did you regularly participate in physical activity during your free time?	1 =daily, 2 = several times a week, 3 = several times a month, 4 = several times a year or less, 5 = never	Delete the sample that answered 5 never participates in sports. 1 and 2 = 1 are frequent participants, 3 and 4 = 0 are not frequent participants	1 = yes, 0 = no
Participation frequency	A30.9 In the past year, did you regularly participate in physical activity during your free time?	1 = daily, 2 = several times a week, 3 = several times a month, 4 = several times a year or less, 5 = never	Delete the sample that answered 5 never participates in sports. 1 = 5 daily, 2 = 4 times a week, 4 = 2 times a year or less, 3 unchanged	5 = Every day, 4 = Several times a week, 3 = Several times a month, 2 = Several times a year or less
Provincial economies	No questions, based on specific provinces where respondents are located and measured in conjunction with the China Statistical Yearbook	Specific province of the respondent	Based on the detailed data of provincial GDP per capita recorded in the 2022 China Statistical Yearbook, provincial GPD per capita is grouped into quintiles and then ranked. Specifically, they are categorized into developed regions, sub-developed regions, medium regions, less developed regions, and underdeveloped regions. The highest GDP per capita is in Beijing, at 183,980 yuan, and the lowest is in Gansu Province, at 41,046 yuan, a difference of 142,934 yuan. Equalized into five equal parts is 28,586.8 yuan each. Calculated: 5 = 41,046–69,632.8 yuan, 1 = 69,632.8–98,219.6 yuan, 2 = 98,219.6–126,806.4 yuan, 3 = 126,806.4–155,393.2 yuan, 4 = 155,393–183,980 yuan	4 = developed, 3 = sub-developed, 2 = medium, 1 = less developed, 5 = underdeveloped
settlement type	No questions, recorded directly by the interviewer	1 = urban, 2 = rural	Unprocessed, using raw data	
Gender	A2.What is your gender?	1 = male, 2 = female	Unprocessed, using raw data	
Ethnicity	A4. Your ethnicity is	1 = Han, 2 = Mongolian, 3 = Manchurian, 4 = Hui, 5 = Tibetan, 6 = Strong, 7 = Vi, 8 = Other	2 = 0, 3 = 0, 4 = 0, 5 = 0, 6 = 0, 7 = 0, 8 = 0.rest unchanged	1 = Han, 0 = national minority
Religious belief	A5.What is your religious belief?	1 = No religion, 11 = Buddhism, 12 = Taoism, 13 = Folk beliefs (worship of Mazu, Kwan Kwong, etc.), 14 = Muslim/Islamic, 15 = Catholic, 16 = Christian, 17 = Orthodox, 18 = Other Christian, 19 = Jewish, 20 = Hindu, 21 = Other	11 = 0, 12 = 0, 13 = 0, 14 = 0, 15 = 0, 16 = 0, 17 = 0, 18 = 0, 19 = 0, 20 = 0, 21 = 0.rest unchanged	1 = no, 0 = yes.
education	A7a.Your current highest level of education:	1 = no education, private school, literacy class, 2= elementary school, 3 = junior high school, 4 = vocational high school, 5 = general high school, 6 = secondary school, 7 = technical school, 8 = university college (adult higher education), 9 = university college (regular higher education), 10 = university bachelor's degree (adult higher education), 11 = university undergraduate degree (regular higher education), 12 = postgraduate student and above, 13 = others	1 = 5, 2 = 1, 3 = 1, 4 = 2, 5 = 3, 6 = 3, 7 = 3, 8 = 3, 9 = 4, 10 = 4, 11 = 4, 12 = 4, 13 = 4	5-no education; 1-Primary education; 2-Secondary education; 3- Upper secondary education; 4-Higher education
BMI	No questions based on “A13. What is your current height in centimeters?” and “A14. What is your current weight in pounds?” The answers to the two questions were calculated	Respondents' specific height (centimeters) and weight (pounds) values were recorded	The specific BMI value was first calculated based on the formula: BMI = (a14/2)/[(a13/100)2]. Then based on the World Health Organization classification criteria, BMI was categorized as “3-under” (<18.5), “1-normal” (18.5–25), and “2-High” (>25)	3 = under, 1 = normal, 2 = high
Health status	A15.You feel that your current health status is:	1 = very unhealthy, 2 = rather unhealthy, 3 = average, 4 = comparatively healthy, 5 = very healthy	1 = 2, rest unchanged	2 = unhealthy, 3 = average, 4 = comparatively healthy, 5 = very healthy
Health issues influence	A16. In the past four weeks, how often has your work or other daily activities been affected by health problems:	1 = always, 2 = often, 3 = sometimes, 4 = rarely, 5 = never	1 = 2, rest unchanged	2 = Frequent, 3 = Sometimes, 4 = Rarely, 5 = Never
depression	A17. In the past four weeks, how often have you felt depressed or down:	1 = always, 2 = often, 3 = sometimes, 4 = rarely, 5 = never	1 = 2, rest unchanged	2 = Frequent, 3 = Sometimes, 4 = Rarely, 5 = Never
Migration	A25.What year did you come to live locally (in your district/county/county)?	The answer recorded the specific year: [____] year. In addition, answer 9996 indicates that the person has lived in the local area only for a short period of time. 9997 indicates that the person has lived in the local area since birth	9997 = 1, other answers = 0	1 = Local, 0 = migrant
Internet access	A28.5 In the past year, your use of the Internet (including cell phone access) was	1 = never, 2 = rarely, 3 = sometimes, 4 = often, 5 = very often	1 = 5, 2 = 1, 3 = 2, 4 = 3, 5 = 4	5 = never, 1 = rarely, 2 = sometimes, 3 = often, 4 = very frequent
Watched competitions	A30. In the past year, did you often go to live sports games in your free time?	1 = daily, 2 = several times a week, 3 = several times a month, 4 = several times a year or less, 5 = never	1 = 4, 2 = 4, 3 = 4, rest unchanged	4 = yes, 5 = no
Social class	A43a. On balance, where do you think you are in society today? On balance, in our current society, where do you personally fit in? (The highest score of “10” represents the top and the lowest score of “1” represents the bottom.)	1 to 10 points	1 and 2 = 1 lower, 3 and 4 = 2 lower middle, 5 and 6 = 3 middle, 7.8.9 and 10 = 4 upper	1 = lower, 2 = lower middle, 3 = middle, 4 = upper
Economic status	A43e.On balance, it appears that your own socio-economic status in this current society belongs to:	1 = upper, 2 = upper middle, 3 = middle, 4 = lower middle, 5 = lower	1 = 2, rest unchanged	2 = upper, 3 = middle, 4 = lower middle, 5 = lower
Overtime work	A53. Did you work more than 1 h in the last week in order to earn an income (including enlisting in the military)?	1 = not engaged in any work for the purpose of obtaining financial income, 2 = paid vacation, study, temporary layoff or seasonal break, etc., 3 = unpaid vacation, study, temporary layoff or seasonal break, etc., 4 = yes, generally [___] hours of work per week	1 = 0, 2 = 0, 3 = 0; the answer to option 4 is filled in with specific working hours in hours. By calculating the true specific working hours, the weekly working hours less than or equal to 40 h are coded as 0 and those greater than 40 h are coded as 1	1 = yes, 0 = no
Household economy	A64.Where does your family's household economic status fall in your locality?	1 = well below average, 2 = below average, 3 = average, 4 = above average, 5 = well above average	5 = 4, rest unchanged	4 = high level, 3 = average, 2 = below average, 1 = well below average
Car	A66. Does your household own a family car?	1 =yes, 2= no	Unprocessed, using raw data	
Children	A68b.How many minor children under the age of 18 do you have?	Specific number of children recorded: [____]	Values greater than or equal to 2 = 2, 0 = 3, rest unchanged	3 = None, 1 = 1, 2 = 2 or above
Spouse	A69.Your current marital status is:	1 = Unmarried, 2 = Cohabiting, 3 = First marriage with spouse, 4 = Remarriage with spouse, 5 = Separated not divorced, 6 = Divorced, 7 = Widowed	2.3.4 and 5 = 1 with spouse; 1.6 and 7 = 0 without spouse	1 = yes, 0 = no
Income	A8a.What was your personal gross income for the whole of last year?	Specific income values in RMB are recorded	Go to logarithm of original data, Income = ln (a8a+1)	Specific values
Age	AA3. What is your date of birth?	Recorded specific year of birth	Age = 2021-a3a	Specific values

#### 2.2.2 Independent variables measurement

Based on the proposed hypothesis—that variables previously identified in the literature as significantly associated with SP are also relevant to Chinese adults—this study incorporates a broad range of predictors that have been empirically validated in prior research. First, existing literature consistently identifies gender and age as key correlates of SP (Oliveira-Brochado et al., 2017; Borgers et al., 2016; Bauman et al., 2012; Amornsriwatanakul et al., 2023; Zasimova, 2022; Downward and Rasciute, 2015; Eberth and Smith, 2010; Charway and Strandbu, 2024; Borgers et al., 2018). In addition, numerous studies have documented significant associations between SP and factors such as regional economic (Zasimova, 2022), settlement type (Zasimova, 2022), ethnicity (Dong et al., 2023), religious belief (Strandbu et al., 2020), education (Oliveira-Brochado et al., 2017), BMI (Oliveira-Brochado et al., 2017), health status (Zasimova, 2022), depression (Lepir and Lakić, 2025), migration (Hallmann et al., 2012), Internet access (Zhong et al., 2022), social class (Oliveira-Brochado et al., 2017), economic status (Eime et al., 2015), working-time (Borgers et al., 2016), household economy (Eime et al., 2015), car (Downward and Rasciute, 2010), children (Zasimova, 2022), spouse (Borgers et al., 2016), and income (Zasimova, 2022).

Finally, lifestyle behaviors that involve exposure to sport, such as frequently watching competitions, have also been shown to significantly influence individual levels of SP (Downward and Riordan, 2007). The specific measures of the independent variables are shown in Table 1.

### 2.3 Samples screening and variables assignment

In accordance with the study's hypotheses and objectives, three dependent variables and a total of 22 independent variables—identified based on prior literature on SP—were included in the analysis. During the variable measurement and coding process using the 2021 CGSS dataset, unusable cases were excluded, including responses marked as “don't know” or “refused to answer.” For example, in response to the height question, 134 participants answered “998” (don't know), and 10 answered “999” (refused). After excluding such cases, a final analytical sample of 5,581 valid observations was obtained. To analyze correlates of whether participation, the full dataset of 5,581 respondents was used. For the analysis of participation frequency and frequent participation, only respondents who reported engaging in SP were included. Samples of individuals who never participated in sports activities were excluded from the analysis, resulting in a final subsample of 3,491 valid cases.

To ensure accurate statistical analysis and model estimation (e.g., chi-square tests), certain response categories with less than 5% representation were merged based on sociological relevance (e.g., vocational high school under the education variable). This also helped reduce potential measurement error caused by inconsistent interpretation of response options among participants. Categorization choices were assigned clear sociological meaning. Additionally, continuous variables with skewed distributions (e.g., the number of minor children) were either recoded into categorical variables or log-transformed to address issues of clustering and sparse observations, thereby improving estimation accuracy. Details on all variable coding are presented in Table 1. All samples screening and variables assignment were conducted using SPSS Statistics for Windows, Version 27.0.

### 2.4 Data analysis

The descriptive statistics were conducted on both the full sample (*N* = 5,581) and the SP subsample (*n* = 3,491) to summarize the sample characteristics. To comprehensively explore the correlates of SP among Chinese adults, this study incorporated three distinct outcome variables. The modeling procedures for each of these outcomes followed a two-step approach, based on the analytical strategy proposed by Victora et al. and supported by prior studies (Amornsriwatanakul et al., 2023; Victora et al., 1997). Specifically, both univariate and backward stepwise regression analyses were employed.

In the first step, the univariate analyses were conducted to examine the association between each independent variable and the outcome variables. For the two binary outcome variables—whether participation and frequent participation—Pearson's chi-square tests were used when the independent variable was categorical, and univariate binary logistic regression was used when the independent variable was numerical. For participation frequency, which is an ordinal categorical outcome, univariate ordinal probit regression was applied. The significance values from the model fit statistics were used to determine whether each independent variable was significantly associated with the outcome. Variables that were significantly associated with the outcome (*p* < 0.05) in the univariate analysis were included in the second-step regression models. This inclusion criterion was consistently applied across all models. Notably, age and gender were retained in all models regardless of significance, as previous literature consistently identifies them as robust correlates of SP (Amornsriwatanakul et al., 2023). Before conducting the second-step modeling, multicollinearity among the independent variables was assessed using Spearman correlation analysis. No severe multicollinearity was detected, as no pairwise correlation coefficient exceeded an absolute value of 0.7. This is one of the commonly used methods for detecting multicollinearity (Chao et al., 2025).

In the second step, all significant variables from the first step were entered into the initial regression model. Based on the criteria of backward stepwise regression, only variables that remained significant (*p* < 0.05) were retained in the subsequent models. Non-significant variables were excluded iteratively until a final model was generated in which all retained variables were significantly associated with the outcome. For the binary outcomes (whether participation and frequent participation), backward binary logistic regression was used. Model fit was assessed using the Hosmer–Lemeshow goodness-of-fit test, with *p*-values greater than 0.05 indicating acceptable model fit. For the ordinal outcome (participation frequency), backward ordinal regression was employed, and model fit was evaluated using the −2 log-likelihood ratio test. All analyses were conducted using SPSS Statistics for Windows, Version 27.0.

## 3 Result

### 3.1 Results of descriptive statistics

Table 2 presents the descriptive statistics for both the full sample and the SP subsample, including all measured variables. In the total sample, 62.6% of respondents reported participating in sports, while 37.4% reported no participation at all. This indicates that the total sample size was 67% higher than the subsample size for SP. A majority of the respondents (73.8%) were from economically underdeveloped or less-developed provinces, whereas only 8.7% were from economically developed provinces. Urban residents accounted for 66.1% of the total sample, nearly double the proportion of rural residents (33.9%). The gender distribution was relatively balanced, with 44.6% male and 55.4% female. Local residents made up 67.6% of the total sample, while migrants accounted for 32.4%. Notably, 83.3% of respondents reported never watching sports competitions, while only 16.7% reported doing so, revealing a large disparity. Regarding socioeconomic status, only 6.4% identified as upper class, while 54.1% belonged to the lower or lower-middle class, and 39.5% to the middle class. Additionally, 42.8% of households reported owning a car, while 57.2% did not, reflecting a relatively even distribution.

**Table 2:** Descriptive statistics of all variables.

**Variable names**	**Category**	**Total sample**		**Subsample of SP**	
		***N*** = **5,581**	**%**	***N*** = **3,491**	**%**
**Dependent variables**					
Whether participation	Yes	3,491	62.6		
	No	2,090	37.4		
Frequent participation	Yes			2,254	64.6
	No			1,237	35.4
Participation frequency	Every day			1,481	42.4
	Several times a week			773	22.1
	Several times a month			624	17.9
	Several times a year or less			613	17.6
**Categorical independent variable (CIV)**					
Provincial economies	Underdeveloped	2,294	41.1	1,383	39.6
	Less developed	1,826	32.7	1,002	28.7
	Medium	600	10.8	434	12.4
	Sub-developed	366	6.6	271	7.8
	Developed	495	8.7	401	11.5
Settlement type	Urban	3,689	66.1	2,602	74.5
	Rural	1,892	33.9	889	25.5
Gender	Male	2,488	44.6	1,594	45.7
	Female	3,093	55.4	1,897	54.3
Ethnicity	National minority	387	6.9	227	6.5
	Han	5,194	93.1	3,264	93.5
Religious belief	Yes	439	7.9	268	7.7
	No	5,142	92.1	3,223	92.3
Education	No education	601	10.8	229	6.6
	Primary education	1,359	24.4	642	18.4
	Secondary education	1,830	32.8	1,128	32.3
	Upper secondary education	1,016	18.2	782	22.4
	Higher education	775	13.9	710	20.3
BMI	Under	398	7.1	198	5.7
	Normal	3,503	62.8	2,231	63.9
	High	1,680	30.1	1,062	30.4
Health status	Unhealthy	1,101	1,101	498	14.3
	Average	1,699	1,699	1,087	31.1
	Comparatively healthy	1,910	1,910	1,338	38.3
	Very healthy	871	871	568	16.3
Health issues influence	Frequent	1,005	18	447	12.8
	Sometimes	888	15.9	534	15.3
	Rarely	1,170	21	811	23.2
	Never	2,518	45.1	1,699	48.7
Depression	Frequent	635	11.4	301	8.6
	Sometimes	1,247	22.3	753	21.6
	Rarely	1,413	25.3	951	27.2
	Never	2,286	41	1,486	42.6
Migration	Migrant	1,808	32.4	1,242	35.6
	Local	3,773	67.6	2,249	64.4
Internet access	Never	1,677	30	748	21.4
	Rarely	410	7.3	247	7.1
	Sometimes	475	8.5	295	8.5
	Often	1,187	21.3	823	23.6
	Very frequent	1,832	32.8	1,378	39.5
Watched competitions	Yes	933	16.7	812	23.3
	No	4,648	83.3	2,679	76.7
Social class	Lower	1,034	18.5	527	15.1
	Lower middle	1,570	28.1	950	27.2
	Middle	2,469	44.2	1,652	47.3
	Upper	508	9.1	362	10.4
Economic status	Upper	356	6.4	266	7.6
	Middle	2,205	39.5	1,485	42.5
	Lower middle	1,728	31	1,117	32
	Lower	1,292	23.1	623	17.8
Overtime work	No	4,000	71.7	2,502	71.7
	Yes	1,581	28.3	989	28.3
Household economy	Well below average.	484	8.7	229	6.6
	Below average	1,912	34.3	1,087	31.1
	Average	2,751	49.3	1,841	52.7
	High level	434	7.8	334	9.6
Car	Yes	2,388	42.8	1,717	49.2
	No	3,193	57.2	1,774	50.8
Children	None	3,822	68.5	2,240	64.2
	1	1,015	18.2	763	21.9
	2 or above	744	13.3	488	14
Spouse	No	806	14.4	454	13
24.6-2.2,-1.3498pt	Yes	4,775	85.6	3,037	87
**Numerical independent variable**	**Category**	**Mean**	**SD**	**Mean**	**SD**
Income (logarithm)	Numerical value	8.24	4.07	8.8	3.87
Age	Numerical value	55.67	14.62	53.94	14.57

Percentages in each group may not total 100 due to rounding.

In the SP subsample—excluding those who never participate in sports −64.6% of respondents were classified as frequent participation, while 35.4% did not meet frequent participation. For participation frequency, 42.4% reported engaging in sport daily, 22.1% several times per week, 17.9% several times per month, and 17.6% only a few times per month. In this subsample, 11.5% of respondents were from economically developed provinces, up from 8.7% in the total sample. The subsample size from economically underdeveloped and less developed provinces together amounted to 68.3%, which is lower compared to the total sample of 73.8%. These findings may suggest higher SP rates in economically developed areas. Urban respondents constituted 74.5% of the subsample, almost three times the rural proportion of 25.5%, a large difference from the total sample. Males accounted for 45.7% and females 54.3%, maintaining a balanced gender distribution similar to the total sample. Regarding sports competitions viewing habits, 76.7% reported never watching competitions, while 23.3% did—a smaller gap compared to the total sample. In terms of socioeconomic status, 7.6% identified as upper class, 49.8% as lower or lower-middle class, and 42.5% as middle class, indicating a relative increase in upper and middle-class representation. Additionally, 49.8% of respondents reported owning a car, compared to 50.2% without, reflecting a slightly higher rate of car ownership than in the total sample. Descriptive statistics for other variables are detailed in Table 2.

### 3.2 Results of the univariate analysis of outcome variables

Table 3 presents the results of the univariate selection process for the three outcome variables, showing only significance levels (*p*). Whether participation (binary outcome): 19 of the 22 candidate variables showed significant associations and were retained for the first backward-stepwise logistic regression. Ethnicity (*p* = 0.101), religion (*p* = 0.498), and overtime work (*p* = 0.997) were excluded due to non-significance. Frequent participation (binary outcome): Six variables failed to reach significance: sex (*p* = 0.531), religion (*p* = 0.234), BMI (*p* = 0.193), self-rated health (*p* = 0.155), family economic status (*p* = 0.114), and personal income (*p* = 0.269). Gender was retained on theoretical grounds, while the other five were excluded, leaving 17 predictors for the backward-stepwise logistic regression. Participation frequency (ordinal outcome): four variables were non-significant: gender (*p* = 0.268), religion (*p* = 0.392), BMI (*p* = 0.225), and personal income (*p* = 0.396). Again, gender was retained based on prior evidence, and the remaining three were excluded, resulting in 19 predictors entering the first backward-stepwise ordinal regression. This rigorous pre-selection ensures that subsequent multivariate models include only variables demonstrating univariate associations with the respective outcomes.

**Table 3:** Results of the univariate analysis of all outcome variables.

**Independent variable**	**P (Whether participation)**	**P (Frequent participation)**	**P (Participation frequency)**
Provincial economies	0.001	0.001	0.001
Settlement type	0.001	0.001	0.001
Gender	0.036	0.531	0.268
Ethnicity	0.101	0.001	0.002
Religious belief	0.498	0.234	0.392
Education	0.001	0.002	0.001
BMI	0.001	0.193	0.225
Health status	0.001	0.155	0.047
Health issues influence	0.001	0.001	0.001
Depression	0.001	0.001	0.001
Migration	0.001	0.001	0.001
Internet access	0.001	0.001	0.001
Watched competitions	0.001	0.001	0.001
Social class	0.001	0.001	0.001
Economic status	0.001	0.006	0.001
Overtime work	0.997	0.001	0.001
Household economy	0.001	0.114	0.016
Car	0.001	0.001	0.001
Children	0.001	0.001	0.001
Spouse	0.001	0.003	0.002
Income	0.001	0.269	0.396
Age	0.001	0.001	0.001

P, significance levels.

### 3.3 Results of regression analysis of outcome variables

Table 4 presents the results of the backward stepwise binary logistic regression analysis on the correlates of whether participation, showing regression coefficients (β), standard error (SE) and significance levels (P). Model 1 included the 19 predictors identified in the univariate screening. The Hosmer–Lemeshow test (*p* = 0.07) indicated an adequate fit. Six variables—self-rated health, migration status, household economic region, presence of children, spouse status, and personal income—were not significantly associated with participation (*p* > 0.05) and were removed (with gender retained on theoretical grounds), yielding 13 predictors for Model 2. Model 2 demonstrated good fit (Hosmer–Lemeshow *p* = 0.174), and all predictors except gender remained significant.

**Table 4:** Backward stepwise binary logistic regression of correlates of whether participation.

**Variables**	**Model 1**			**Model 2**		
	**β**	**SE**	***p*-value**	**β**	**SE**	***p*-value**
Provincial economies (ref = underdeveloped)						
Less developed	−0.048	0.072	0.498	−0.045	0.071	0.527
Medium	0.367	0.114	0.001	0.377	0.113	0.001
Sub-developed	0.552	0.14	<0.001	0.58	0.14	<0.001
Developed	0.354	0.141	0.012	0.381	0.138	0.006
Settlement type (ref = rural)	0.448	0.07	<0.001	0.476	0.068	<0.001
Gender (ref = female)	−0.053	0.069	0.442	−0.019	0.066	0.773
Education (ref = no education)						
Primary education	0.254	0.109	0.02	0.27	0.108	0.013
Secondary education	0.572	0.112	<0.001	0.599	0.111	<0.001
Upper secondary education	1.046	0.133	<0.001	1.091	0.131	<0.001
Higher education	1.888	0.179	<0.001	1.98	0.177	<0.001
BMI (ref = under)						
Normal	0.424	0.122	0.001	0.448	0.122	<0.001
High	0.395	0.129	0.002	0.412	0.129	0.001
Health status (ref = very healthy)				EXC		
Unhealthy	−0.007	0.13	0.955			
Average	0.111	0.106	0.296			
Comparatively healthy	0.189	0.099	0.056			
Health issues influence (ref = never)						
Frequent	−0.232	0.114	0.041	−0.289	0.1	0.004
Sometimes	−0.019	0.103	0.855	−0.026	0.098	0.791
Rarely	0.058	0.093	0.53	0.086	0.091	0.347
Depression (ref = never)						
Frequent	0.052	0.12	0.662	0.022	0.117	0.852
Sometimes	0.162	0.091	0.074	0.151	0.089	0.091
Rarely	0.234	0.088	0.008	0.237	0.088	0.007
Migration (ref = Local)	0.025	0.072	0.729	EXC		
Internet access (ref = never)						
Rarely	0.331	0.125	0.008	0.353	0.125	0.005
Sometimes	0.399	0.123	0.001	0.418	0.122	0.001
Often	0.364	0.102	<0.001	0.385	0.101	<0.001
Very frequent	0.533	0.1	<0.001	0.561	0.1	<0.001
Watched competitions (ref = No)	1.333	0.109	<0.001	1.321	0.109	<0.001
Social class (ref = upper)						
Lower	−0.287	0.149	0.054	−0.302	0.148	0.041
Lower middle	−0.318	0.137	0.02	−0.326	0.137	0.017
Middle	−0.239	0.124	0.054	−0.244	0.124	0.049
Economic status (ref = lower)						
Upper	0.479	0.174	0.006	0.54	0.168	0.001
Middle	0.283	0.103	0.006	0.326	0.098	0.001
Lower middle	0.229	0.092	0.013	0.253	0.091	0.005
Household economy (ref = high level)				EXC		
Well below average	−0.072	0.179	0.686			
Below average	−0.143	0.149	0.337			
Average	−0.054	0.14	0.7			
Car (ref = No)	0.193	0.069	0.005	0.199	0.068	0.003
Children (ref = none)				EXC		
1	0.174	0.107	0.103			
2 or above	−0.17	0.116	0.141			
Spouse (ref = yes)	0.045	0.091	0.621	EXC		
Age	0.005	0.004	0.166	0.006	0.003	0.049
Income	0.015	0.008	0.074	EXC		
**Hosmer–Lemeshow test**			0.07			0.174

^a^95%CI.

^b^Ref = reference category.

^c^EXC = excluded from the model because it did not meet the inclusion criteria for the analysis.

In the final Model 2, compared with adults in underdeveloped provinces, those in developed (β = 0.381, *p* = 0.006), sub-developed (β = 0.580, *p* < 0.001), and medium provinces (β = 0.377, *p* = 0.001) had higher odds of SP. Urban residency was positively associated (β = 0.476, *p* < 0.001), while gender was not significant (*p* = 0.773). Educational attainment showed a significant dose effect: primary (β = 0.270, *p* = 0.013), secondary (β = 0.599, *p* < 0.001), upper secondary (β = 1.091, *p* < 0.001), and higher education (β = 1.980, *p* < 0.001) all participated in sport to a greater extent than uneducated adults. And the β-value increases with the level of education, indicating that the higher the level of education, the higher the level of SP. Adults with normal (β = 0.448, *p* < 0.001) and high (β = 0.412, *p* = 0.001) BMI were more active in SP compared to adults with under-normal BMI. Frequent health issues reduced participation likelihood (β = −0.289, *p* = 0.004), whereas rarely depressive symptoms increased it (β = 0.237, *p* = 0.007). In terms of Internet access, adults who used the Internet rarely (β = 0.353, *p* = 0.005), sometimes (β = 0.418, *p* = 0.001), often (β = 0.385, *p* < 0.001), and very often (β = 0.561, *p* < 0.001) were more involved in sport compared to those who never used the Internet. Those who used it most frequently had the largest β values, indicating that the level of participation was the highest. Watching competitions was a strong predictor (β = 1.321, *p* < 0.001). In terms of social class: lower (β = −0.302, *p* = 0.041), lower-middle (β = −0.326, *p* = 0.017), and middle (β = −0.244, *p* = 0.049) had lower odds in SP than the upper class. Personal economic status exhibited a positive gradient: lower-middle (β = 0.253, *p* = 0.005), middle (β = 0.326, *p* = 0.001), and upper (β = 0.540, *p* = 0.001) status each increased odds relative to the lower. Adults with a car in the household (β =0.199, *p* =0.003) were more involved in sports than those without a car. Finally, age was positively associated with participation (β = 0.006, *p* = 0.049).

Table 5 presents the results of the backward stepwise binary logistic regression analysis on the correlates of frequent participation, showing regression coefficients (β), standard error (SE) and significance levels (*p*). Seventeen predictors identified in the univariate screening were entered into Model 3. The Hosmer–Lemeshow test (*p* = 0.684) confirmed adequate fit. Nine variables—ethnicity, education, Internet access, watched competitions, social class, economic status, overtime work, car, and spouse—were not significantly associated with frequent participation, and were thus excluded. Eight remaining predictors proceeded to Model 4, which retained only those with *p* < 0.05 (with gender again preserved on theoretical grounds) and also demonstrated good fit (Hosmer–Lemeshow *p* = 0.552).

**Table 5:** Backward stepwise binary logistic regression of correlates of frequent participation.

**Variables**	**Model 3**			**Model 4**		
	β	**SE**	***p*** **-value**	β	**SE**	***p*** **-value**
Provincial economies (ref = underdeveloped)						
Less developed	0.346	0.095	<0.001	0.334	0.092	<0.001
Medium	0.06	0.125	0.632	0.076	0.123	0.533
Sub-developed	0.306	0.154	0.047	0.3	0.152	0.049
Developed	0.109	0.139	0.432	0.186	0.134	0.166
Settlement type (ref = rural)	0.197	0.095	0.038	0.281	0.09	0.002
Gender (ref = female)	−0.048	0.081	0.554	−0.028	0.077	0.717
Ethnicity (ref = Han)	−0.096	0.155	0.535	EXC		
Education (ref = no education)				EXC		
Primary education	−0.176	0.176	0.318			
Secondary education	0.148	0.173	0.394			
Upper secondary education	0.323	0.187	0.083			
Higher education	0.183	0.197	0.351			
Health issues influence (ref = never)						
Frequent	−0.195	0.141	0.166	−0.224	0.139	0.107
Sometimes	−0.466	0.119	<0.001	−0.488	0.118	<0.001
Rarely	−0.361	0.103	<0.001	−0.372	0.103	<0.001
Depression (ref = never)			<0.001			
Frequent	0.002	0.163	0.991	−0.03	0.16	0.852
Sometimes	−0.386	0.109	<0.001	−0.405	0.107	<0.001
Rarely	−0.601	0.102	<0.001	−0.615	0.101	<0.001
Migration (ref = Local)	0.226	0.085	0.008	0.227	0.085	0.007
Internet access (ref = Never)				EXC		
Rarely	−0.146	0.17	0.389			
Sometimes	−0.158	0.161	0.326			
Often	0.078	0.137	0.567			
Very frequent	0.089	0.134	0.508			
Watched competitions (ref = No)	−0.092	0.09	0.305	EXC		
Social class (ref = upper)				EXC		
Lower	−0.014	0.182	0.938			
Lower middle	−0.164	0.158	0.3			
Middle	−0.171	0.141	0.224			
Economic status (ref = lower)				EXC		
Upper	0.223	0.195	0.254			
Middle	0.157	0.129	0.224			
Lower middle	0.034	0.121	0.778			
Overtime work (ref = yes)	0.041	0.089	0.645	EXC		
Car (ref = No)	−0.114	0.082	0.167	EXC		
Children (ref = none)						
1	−0.285	0.117	0.015	−0.307	0.115	0.008
2 or above	−0.394	0.134	0.003	−0.43	0.133	0.001
Spouse (ref = yes)	0.03	0.121	0.806	EXC		
Age	0.033	0.005	<0.001	0.03	0.004	<0.001
**Hosmer–Lemeshow test**			0.684			0.522

^a^95%CI.

^b^Ref = reference category.

^c^EXC = excluded from the model because it did not meet the inclusion criteria for the analysis.

In the final Model 4, compared to underdeveloped provinces, adults in sub-developed provinces had higher odds of frequent participation (β = 0.300, *p* = 0.049). Urban residency was positively associated (β = 0.281, *p* = 0.002), whereas gender remained non-significant (*p* = 0.717). Health issues reduced the likelihood of frequent participation: those “rarely” (β = −0.372, *p* < 0.001) or “sometimes” (β = −0.488, *p* < 0.001) affected participated less than those never affected. Depression also had a negative effect: “rarely” (β = −0.615, *p* < 0.001) and “sometimes” depressed individuals (β = −0.405, *p* < 0.001) were less likely to meet frequent participation than never-depressed peers. In terms of migration, migrants (non-locals) showed higher frequent participation (β = 0.227, *p* = 0.007). Relative to adults with no minor children, having 1 (β = −0.307, *p* = 0.008) and 2 or above (β = −0.43, *p* = 0.001) minor children was a significant negative influence on frequent participation. And adults with 2 or above minor children had lower β values than adults with only 1 minor child, indicating less frequent participation. Finally, age was positively associated (β = 0.030, *p* < 0.001), indicating that older adults were more likely to meet frequent participation.

Table 6 presents the results of the backward stepwise ordinal regression analysis of the correlates of participation frequency, showing the regression coefficients (β), standard errors (SE), and levels of significance (*p*). Model 6 in Table 6, the final ordered regression for participation frequency, demonstrated improved fit (−2 log likelihood= 8,192.639 vs. 8,615.532 in Model 5). Eight predictors retained significance (gender retained on theoretical grounds), while 12 in Model 5—ethnicity, education, health status, Internet access, watched competitions, social class, economic status, overtime work, household economy, car, and spouse —were excluded.

**Table 6:** Backward stepwise ordered regression of correlates of participation frequency.

**Variables**	**Model 5**			**Model 6**		
	**β**	**SE**	***p*-value**	**β**	**SE**	***p*-value**
**Dependent variable (ref** = **daily)**						
Several times a year or less	0.032	0.217	0.882	−0.025	0.127	0.843
Several times a month	0.635	0.217	0.003	0.573	0.127	<0.001
Several times a week	1.266	0.218	<0.001	1.199	0.127	<0.001
**Independent variable**						
Provincial economies (ref = underdeveloped)						
Less developed	0.175	0.048	<0.001	0.175	0.047	<0.001
Medium	0.015	0.064	0.819	0.023	0.062	0.712
Sub-developed	0.205	0.077	0.008	0.197	0.076	0.01
Developed	0.166	0.07	0.018	0.199	0.068	0.003
Settlement type (ref = rural)	0.105	0.049	0.032	0.147	0.046	0.002
Gender (ref = female)	−0.003	0.041	0.938	0.004	0.039	0.927
Ethnicity (ref = Han)	−0.02	0.08	0.804	EXC		
Education (ref = no education)				EXC		
Primary education	−0.11	0.088	0.212			
Secondary education	0.094	0.087	0.281			
Upper secondary education	0.148	0.094	0.114			
Higher education	0.062	0.1	0.536			
Health status (ref = very healthy)				EXC		
Unhealthy	0.016	0.086	0.852			
Average	−0.017	0.064	0.789			
Comparatively healthy	0	0.058	0.995			
Health issues influence (ref = never)						
Frequent	−0.134	0.079	0.088	−0.133	0.07	0.056
Sometimes	−0.291	0.063	<0.001	−0.303	0.061	<0.001
Rarely	−0.26	0.053	<0.001	−0.268	0.052	<0.001
Depression (ref = never)						
Frequent	0.015	0.082	0.86	0.003	0.08	0.969
Sometimes	−0.154	0.056	0.006	−0.17	0.055	0.002
Rarely	−0.289	0.052	<0.001	−0.301	0.052	<0.001
Migration (ref = Local)	0.161	0.043	<0.001	0.166	0.043	<0.001
Internet access (ref = Never)				EXC		
Rarely	−0.158	0.085	0.064			
Sometimes	−0.113	0.082	0.169			
Often	−0.004	0.068	0.954			
Very frequent	0.028	0.066	0.673			
Watched competitions (ref = No)	−0.039	0.046	0.397	EXC		
Social class (ref = upper)				EXC		
Lower	0.028	0.092	0.76			
Lower middle	−0.108	0.079	0.174			
Middle	−0.061	0.07	0.382			
Economic status (ref = lower)				EXC		
Upper	0.126	0.101	0.212			
Middle	0.091	0.068	0.183			
Lower middle	0.03	0.063	0.628			
Overtime work (ref = yes)	0.072	0.046	0.117	EXC		
Household economy (ref = high level)				EXC		
Well below average	0.06	0.11	0.587			
Below average	−0.048	0.08	0.553			
Average	−0.025	0.072	0.733			
Car (ref = No)	−0.043	0.042	0.309	EXC		
Children (ref = none)						
1	−0.14	0.061	0.021	−0.156	0.06	0.009
2 or above	−0.164	0.07	0.019	−0.176	0.069	0.011
Spouse (ref = yes)	−0.017	0.06	0.775	EXC		
Age	0.02	0.002	<0.001	0.019	0.002	<0.001
**−2 log likelihood**	8,615.532			8,192.639		

^a^95%CI.

^b^Ref = reference category.

^c^EXC = excluded from the model because it did not meet the inclusion criteria for the analysis.

In Model 6: provincial economies: adults in developed (β = 0.199, *p* = 0.003), sub-developed (β = 0.197, *p* = 0.010), and less developed provinces (β = 0.175, *p* < 0.001) reported higher practice frequency than those in underdeveloped provinces. Urban residence: positively associated with frequency (β = 0.147, *p* = 0.002). gender: non-significant (*p* = 0.927). Health issues: “rarely” (β = −0.268, *p* < 0.001) and “sometimes” affected (β = −0.303, *p* < 0.001) adults practiced less often than those never affected. Depression: “rarely” (β = −0.301, *p* < 0.001) and “sometimes” depressed (β = −0.170, *p* = 0.002) adults had lower frequency than never-depressed peers. In terms of migration, migrants practiced more frequently (β = 0.166, *p* < 0.001). Children: one child (β = −0.156, *p* = 0.009) and 2 or above children (β = −0.176, *p* = 0.011) each reduced frequency, with a stronger effect for adults having 2 or above children. Age: positively associated (β = 0.019, *p* < 0.001), indicating that practice frequency increases with age.

## 4 Discussion

This study leveraged nationally representative CGSS data to examine the correlates of Chinese adults' SP, operationalized in three ways: (1) whether participation, (2) participation frequency, and (3) frequent participation. We selected 22 predictors validated in prior SP research. Our research found that: (1) these established correlates also significantly influence Chinese adults' SP, though effect sizes vary; and (2) the correlates of participation frequency and frequent participation are largely congruent, but they differed substantially from those associated with the correlates of whether participation. The primary objective of this study was to identify the factors influencing SP among Chinese adults to provide an objective basis and direction for the development of targeted intervention measures. Therefore, the discussion of the results focuses mainly on the variables that show a significant correlation with outcome variables, aiming to inform more effective public health policies and interventions.

A key and novel finding of this study pertains to the association between age and SP. Contrary to a substantial body of prior research (Oliveira-Brochado et al., 2017; Borgers et al., 2016; Downward and Rasciute, 2015; Eberth and Smith, 2010), our results demonstrate that age was significantly and positively associated with all three outcome variables. Although existing literature indicates that SP declines with increasing age among adults globally (Crossman et al., 2024), our findings suggest an opposite trend among Chinese adults. One possible explanation for this discrepancy is the limited availability of time and energy for younger adults in China to engage in SP. Data from the China National Time Use Survey indicate that younger adults allocate a significant portion of their time to education, childcare, and skills development. For instance, Chinese residents aged 25–34 years spend the most time caring for children, averaging 1 h and 16 min per day (National Bureau of Statistics, 2019). Meanwhile, in terms of time spent on learning enhancement, residents aged 20–24, it was 1 h and 38 min (National Bureau of Statistics, 2019). As individuals age, particularly after their children reach adulthood and their careers become more stable, they generally experience an increase in personal leisure time. This shift likely facilitates greater SP, leading to the observed positive association between age and SP among Chinese adults. These findings have important public health implications. They suggest that interventions aimed at promoting SP among younger adults in China should consider the time constraints imposed by childcare and professional development responsibilities. Flexible and accessible exercise programs that integrate with daily routines may be particularly beneficial for this age group. Moreover, age-specific strategies that acknowledge life course transitions could enhance the effectiveness of national SP promotion initiatives.

The association between gender and SP in this study also diverges from much of the existing literature (Oliveira-Brochado et al., 2017; Zasimova, 2022; Downward and Rasciute, 2015; Eberth and Smith, 2010; Charway and Strandbu, 2024; Borgers et al., 2018). Intuitively, men are presumed to be more likely than women to engage in SP. However, in our analysis, gender was not a significant predictor in any of the regression models. A plausible explanation for this finding lies in the broad definition of SP adopted in this study, which did not differentiate between specific types of activities. Previous research has demonstrated that men are generally more inclined toward strength training, while women more frequently engage in aerobic and flexibility-focused exercises (Nuzzo and Deaner, 2023). Consequently, when diverse forms of SP are aggregated without distinction, the types of activities included in the overall sample may balance out across genders, resulting in comparable levels of participation between male and female. These results suggest that gender differences in SP may be highly dependent on the definition and categorization of SP. Future research and public health interventions should consider disaggregating activity types to more accurately capture and address gender-specific patterns in SP.

The findings regarding regional economic status are consistent with previous literature (Eime et al., 2015; Boone-Heinonen et al., 2011). In all three final models analyzing the outcome variables, variable terms related to provincial economies consistently showed significant positive associations. Specifically, for both whether participation and participation frequency, three levels of economic status exhibited significant positive effects. Although the influence of provincial economies weakened in the model for frequent participation—with only sub-developed provinces showing a significant positive association—the overall trend indicates that better economic conditions in provincial regions substantially promote SP. These findings highlight the importance of addressing regional disparities in economic resources when designing and implementing SP promotion strategies at the population level.

The findings regarding education are consistent with prior research (Oliveira-Brochado et al., 2017; Zasimova, 2022). Educational level was significantly and positively associated with whether participation, indicating that individuals with higher levels of education were more likely to engage in SP. Previous studies have suggested that participation in sports during the student years is positively associated with greater SP levels in adulthood (Ramer et al., 2025). Moreover, fostering enjoyment of SP during adolescence has been shown to shape more favorable participation behaviors in early adulthood (Ramer et al., 2025). These mechanisms may partially explain the observed positive relationship between higher educational attainment and greater likelihood of participating in SP. However, education was not significantly associated with participation frequency and frequent participation in our models and is therefore not further discussed in relation to these outcomes.

The settlement type was significantly associated with all three outcome variables. Specifically, urban residents were more likely to participate in SP and more frequently than rural residents. This finding is consistent with previous studies that have highlighted the more active SP observed in urban areas (Eime et al., 2015; Zasimova, 2022; An and Zheng, 2014). Urban areas typically benefit from more developed economies and better infrastructure, including access to superior sports facilities, which can facilitate higher levels of SP. Additionally, research has shown that the effectiveness of national sports policies in rural areas is often limited (An and Zheng, 2014), which contributes significantly to the lower levels of SP observed in rural regions. These factors collectively underscore the need for targeted policy interventions that address the disparities in SP between urban and rural areas.

The health issues influence variable was significantly associated with all three outcome variables. Consistent with a wealth of existing literature, individuals affected by health problems were found to have limited engagement in SP (Zasimova, 2022; Downward and Rasciute, 2015; Eberth and Smith, 2010; Downward and Rasciute, 2010). The rationale behind this finding is straightforward: when health issues become severe enough to interfere with daily life, individuals' physical capabilities are often compromised, making it difficult or even impossible for them to meet the physical demands of regular exercise. These health-related barriers can include chronic illnesses, physical disabilities, or other medical conditions that restrict mobility or energy levels, thereby hindering SP (Warms et al., 2007; Vader et al., 2021; Rasinaho et al., 2007). These findings emphasize the need for public health strategies that consider the unique needs of individuals with health problems, such as tailored exercise programs that accommodate specific health conditions or the development of supportive environments that promote SP for those with limited physical abilities.

An interesting finding emerged from the analysis of psychological problems. Rarely depression was significantly and positively correlated with whether participation. Individuals experiencing rarely psychological issues may turn to SP as a means to improve their mood and cope with stress. This may be due to the fact that physical exercise can provide a mild alleviation of psychological distress (Piercy et al., 2018; Young and Block, 2023). However, when it comes to participation frequency and frequent participation, all indicators of depressions were negatively correlated. This suggests that while minor psychological problems may encourage initial SP, as long as there are any psychological problems still discourage sustained SP. Psychological problems are often accompanied by symptoms such as insomnia, early awakening, and insufficient sleep, which lead to persistent fatigue and lower energy levels (Laskemoen et al., 2019). These sleep disturbances can significantly reduce the likelihood of engaging in regular SP, thereby decreasing participation frequency (Fei et al., 2024). Moreover, psychological issues can also lead to a decline in interest and a lack of self-confidence (Xia et al., 2025), which further diminishes an individual's motivation to participate in SP. These factors combined contribute to a negative impact on the frequency. In summary, while individuals with mild psychological issues may use SP as a temporary relief for their mental state, they are unlikely to sustain high-frequency participation.

The “Internet access” variable was significantly positively correlated with whether participation, which aligns with findings from previous research (Zhong et al., 2022). One possible explanation for this is that the internet provides access to a wide array of sports-related information, which can enhance health awareness and encourage higher levels of SP. However, the “Internet access” variable was not significantly correlated with participation frequency or frequent participation. This suggests that while the internet may play a role in encouraging individuals to engage in SP, it does not necessarily translate into sustained or frequent participation.

Watching competitions was positively correlated with whether participation. This supports existing studies, which suggest that incorporating sports-related content into one's lifestyle can foster greater engagement in SP (Downward and Riordan, 2007). However, whether watching competitions was not significantly correlated with participation frequency and frequent participation.

Regarding social class, the study found that adults belonging to the lower, lower-middle, and middle social classes participated in sports significantly less than those from the upper class. This finding aligns with existing literature (Oliveira-Brochado et al., 2017), which consistently reports that individuals from higher social strata are more likely to engage in sports activities than those from lower strata. One possible explanation is that people from lower social classes may have less awareness of the health benefits associated with SP. In contrast, individuals from higher social classes are more likely to recognize the personal advantages of maintaining good physical fitness, which can be instrumental in sustaining their social status. Notably, social class was not significantly associated with participation frequency or frequent participation.

Income was not significantly associated with SP, a finding consistent with previous literature (Hallmann et al., 2012). However, individual economic status showed a significant positive association with whether participation, which aligns with earlier studies (Eime et al., 2015, 2013). This relationship likely reflects the fact that individuals with higher economic status are better able to overcome financial barriers to participating in sports. It is important to distinguish between personal income and economic status, as they represent fundamentally different concepts. For some individuals, a low or even non-existent current income does not necessarily imply low economic status, as they may have inherited family wealth or accumulated substantial assets earlier in life. Conversely, individuals with high incomes may still have a low economic status if burdened by substantial debts, such as mortgages or car loans. Therefore, individual economic status is a more accurate and comprehensive indicator of one's real financial situation than income alone. Finally, it is worth noting that individual economic status was not significantly associated with participation frequency and frequent participation.

The presence of a car also has a positive effect on promoting SP, which is consistent with findings reported in the literature (Downward and Rasciute, 2010). Owning a car can enhance an individual's accessibility to sports facilities, thereby increasing the likelihood of participating in sports activities. However, cars not have a significant influence on participation frequency or frequent participation.

Adults with normal or high BMI levels demonstrated higher rates of SP compared to those with a BMI below the normal range, which contrasts with some previous research findings (Oliveira-Brochado et al., 2017; Eberth and Smith, 2010). A plausible explanation is that weight management serves as an important motivation for engaging in SP (Deelen et al., 2018; Mutter and Pawlowski, 2014; Gucciardi and Jackson, 2015). Adults with normal or overweight BMI levels may participate in sports to maintain or restore a healthy body weight, whereas individuals with low BMI may lack such motivation. It is particularly noteworthy that in the experimental results of “Whether participation”, the regression coefficient for individuals of normal weight was the highest. This may indicate a non-linear relationship between BMI and this outcome variable, but this requires further data analysis in the future to substantiate. Additionally, BMI was not significantly associated with participation frequency or frequent participation.

This study found that migrants exhibited higher participation frequency and a greater likelihood of frequent participation compared to local residents, a finding that contrasts with some previous literature (Hallmann et al., 2012; Wicker et al., 2013). A possible explanation lies in the motivational factors driving participation. Prior studies have highlighted that social interaction is a major motivator for engaging in sports (Oliveira-Brochado et al., 2017; Borgers et al., 2018). Moreover, research has explicitly pointed out that participation in sports can serve as an important mediator of social inclusion and belonging (Young and Block, 2023). Therefore, migrants may participate more frequently in sports activities as a means to enhance their sense of social belonging and integrate into the local community. Notably, migration status was not significantly associated with whether participation.

The presence of minor children was found to have a negative impact on both participation frequency and the likelihood of frequent participation. Regression results further indicated that the negative effect intensified as the number of minor children increased. This finding is consistent with many previous studies and reports (Oliveira-Brochado et al., 2017; Zasimova, 2022; Harris et al., 2017). It is well-established that the presence of minor children demands substantial time and effort from adults in fulfilling family responsibilities. For example, activities related to children's education and daily care inevitably consume significant personal time (National Bureau of Statistics, 2019), thereby markedly reducing adults' ability to maintain frequent participation. Notably, the presence of minor children was not significantly associated with whether participation.

## 5 Strengths

This study has several notable strengths. First, it provides a comprehensive analysis of the correlates of SP among Chinese adults using nationally representative data from the Chinese General Social Survey (CGSS), which enhances the generalizability of the findings. Second, a wide range of independent variables validated in prior literature were incorporated, together with three distinct outcome variables—whether participation, participation frequency, and frequent participation—allowing for a nuanced and differentiated examination of SP behavior.

Importantly, this study offers several innovative contributions. It demonstrates that although the correlates of participation frequency and frequent participation are largely similar, they differ substantially from those associated with whether participation, highlighting the importance of distinguishing between different dimensions of SP. A particularly novel finding is that SP among Chinese adults increases with age, which contrasts with much of the existing literature and underscores the role of contextual and cultural factors. In addition, the study finds that individuals with mild psychological problems are more likely to engage in SP, although their participation frequency remains relatively low, providing new insights into the complex relationship between mental health and SP. Collectively, these strengths contribute to a more comprehensive understanding of SP among Chinese adults and provide a solid empirical basis for future research and intervention development.

## 6 Limitations and future research directions

Despite the strengths of this study, several limitations should be acknowledged, which also indicate directions for future research. First, although a wide range of variables was included, the selection of variables was not explicitly guided by a comprehensive sociological or behavioral framework. For example, the ecological model was not incorporated at the study design stage, which may limit the multilevel interpretation of the findings. Future research could integrate established theoretical frameworks, such as the ecological model, to better structure variable selection and enhance the explanatory depth of sports participation behavior.

Second, this study adopts a cross-sectional design, which precludes causal inference and restricts the analysis to identifying correlates of sports participation outcomes. In addition, the observational nature of the data limits the ability to examine dynamic or bidirectional relationships among variables, such as the reciprocal association between health status and sports participation. Longitudinal designs or intervention-based studies are therefore needed to strengthen causal inference and explore temporal relationships.

Third, all variables were derived from self-reported data, which may introduce recall bias and social desirability bias, potentially affecting the accuracy of the measurements. Moreover, although the CGSS is a nationally representative survey, certain subpopulations—such as individuals with severe physical disabilities, institutionalized populations, or those with extremely poor health—may be underrepresented, which may limit the generalizability of the findings to all adult populations.

Fourth, while this study incorporated a broad set of sociodemographic, health-related, and social factors, unmeasured confounding cannot be ruled out. Other potentially relevant influences, including environmental characteristics, cultural norms, or individual motivational factors, were not available in the dataset and may have influenced sports participation behaviors.

Finally, the CGSS dataset lacks detailed information on physical activity characteristics, such as activity intensity, duration, and specific types of sports activities. Emerging evidence suggests that the distribution of activity intensity may be more strongly associated with health outcomes than participation frequency alone (Schwendinger et al., 2025). Therefore, future studies may benefit from incorporating participation intensity as an outcome variable, using measures such as metabolic equivalent tasks (METs), to provide more comprehensive assessments of sports participation and its health implications.

## 7 Conclusion

In conclusion, this study provides a comprehensive and differentiated analysis of the correlates of sports participation among Chinese adults based on nationally representative survey data. By distinguishing between whether participation, participation frequency, and frequent participation, the study reveals both shared and distinct correlates across different dimensions of SP.

Based on the findings regarding correlates of SP, future efforts aimed at increasing overall participation rates among Chinese adults should prioritize interventions targeting specific subgroups. These include adults residing in economically underdeveloped provinces, rural areas, those with lower educational attainment, individuals with a BMI below the normal range, adults from lower social classes, those affected by health problems, individuals with low levels of internet use, those who do not watch sports competitions, individuals with lower personal economic status, adults without access to a household car, and younger adults. Specific strategies may involve strengthening the implementation of sports policies in underdeveloped provinces and rural areas, expanding sports facilities in these regions, improving the physical health of adults affected by health problems, raising the overall educational level of the population, promoting internet accessibility and usage, addressing malnutrition among adults with low BMI through dietary interventions, enhancing sports viewership rates, and increasing leisure time while reducing work-related stress among younger adults. Finally, promoting sports participation as a way to relieve psychological problems to people with mild psychological problems is also likely to be an effective way to increase sports participation rates.

The correlates identified for participation frequency and frequent participation were largely consistent. Therefore, future interventions aimed at enhancing participation frequency and the rate of meeting frequent participation among Chinese adults already engaged in sports should focus on adults residing in economically underdeveloped provinces, rural areas, those experiencing health problems, individuals with mental health issues, local (non-migrant) residents, adults with minor children, and younger adults. Potential measures include establishing community-based mental health support centers, promoting SP policies in older urban districts, alleviating the burden of caregiving and education responsibilities for minor children, and the reducing of the stress of social survival and living. Additionally, fostering family-based SP models, where adults and minors engage in physical activities together, may represent an effective strategy to improve both participation frequency and achievement of recommended activity levels.

Overall, this study provides an important empirical foundation for designing integrated strategies to promote sports participation and improve population health in China.

## Data Availability

All relevant data are included in the supplementary materials of this article. Further inquiries can be directed to the corresponding author/s.
